# A phase-space beam position monitor for synchrotron radiation

**DOI:** 10.1107/S1600577515007390

**Published:** 2015-06-25

**Authors:** Nazanin Samadi, Bassey Bassey, Mercedes Martinson, George Belev, Les Dallin, Mark de Jong, Dean Chapman

**Affiliations:** a Biomedical Engineering, University of Saskatchewan, 107 Wiggins Road, Saskatoon, SK, Canada S7N 5E5; b Physics and Engineering Physics, University of Saskatchewan, 116 Science Place, Saskatoon, SK, Canada S7N 5E2; c Canadian Light Source, 44 Innovation Boulevard, Saskatoon, SK, Canada S7N 2V3; d Anatomy and Cell Biology, University of Saskatchewan, 107 Wiggins Road, Saskatoon, SK, Canada S7N 5E5

**Keywords:** beam position monitor, X-ray diffraction, *K*-edge absorption, phase space

## Abstract

The stability of the photon beam position on synchrotron beamlines is critical for most if not all synchrotron radiation experiments. The position of the beam at the experiment or optical element location is set by the position and angle of the electron beam source as it traverses the magnetic field of the bend-magnet or insertion device. Thus an ideal photon beam monitor would be able to simultaneously measure the photon beam’s position and angle, and thus infer the electron beam’s position in phase space. X-ray diffraction is commonly used to prepare monochromatic beams on X-ray beamlines usually in the form of a double-crystal monochromator. Diffraction couples the photon wavelength or energy to the incident angle on the lattice planes within the crystal. The beam from such a monochromator will contain a spread of energies due to the vertical divergence of the photon beam from the source. This range of energies can easily cover the absorption edge of a filter element such as iodine at 33.17 keV. A vertical profile measurement of the photon beam footprint with and without the filter can be used to determine the vertical centroid position and angle of the photon beam. In the measurements described here an imaging detector is used to measure these vertical profiles with an iodine filter that horizontally covers part of the monochromatic beam. The goal was to investigate the use of a combined monochromator, filter and detector as a phase-space beam position monitor. The system was tested for sensitivity to position and angle under a number of synchrotron operating conditions, such as normal operations and special operating modes where the photon beam is intentionally altered in position and angle at the source point. The results are comparable with other methods of beam position measurement and indicate that such a system is feasible in situations where part of the synchrotron beam can be used for the phase-space measurement.

## Abstract 2

A system has been developed to measure the vertical position and angle of the electron beam at a single location from a synchrotron source. The system uses a monochromator tuned to the absorption edge of a contrast material and has a sensitivity comparable with other beam position monitors.

## 1. Introduction   

The trajectory of a photon beam can be determined if both the position and angle can be found at some point along the propagation direction. From knowledge of this beam’s position and angle or its position in phase space, it is possible to transform back to the source and determine the source co­ordinates in phase space if the optics in the intervening locations and their effects are known.

This paper describes a method to simultaneously measure the photon beam position and angle at one location. The system was developed at the Canadian Light Source (CLS) on the Biomedical Imaging and Therapy (BMIT) bend-magnet beamline 05B1-1 (BM).

The CLS is a third-generation synchrotron facility with a low electron beam emittance (∊_*x*_ = 18 nm rad, ∊_*y*_ = 0.10 nm rad). Beam instability, be it electron or photon beam, can be a concern especially to the third-generation facilities. It has adverse effects on the required low electron beam emittance, effective brilliance of the synchrotron radiation, and experiments performed at the experimental stations (Brefeld, 1989 ▸; Haga *et al.*, 2000 ▸; Galimberti & Borghes, 2004 ▸; Rehm, 2013 ▸). The causes of beam instability and the methods of reduction have been widely studied (Hettel, 1989 ▸; Bocchetta, 1996 ▸; Farvacque, 1996 ▸; Hettel, 2002 ▸), and the drive to ensure and maintain a steady beam has led to the development of different types of beam monitors (Billing, 1988 ▸; Izumi *et al.*, 1989 ▸; Johnson & Oversluizen, 1989 ▸; van Silfhout, 1999 ▸; Alkire *et al.*, 2000 ▸; Kyele *et al.*, 2005 ▸; Bunk *et al.*, 2005 ▸; Bergonzo *et al.*, 2006 ▸; Ilinski *et al.*, 2007 ▸; Tucoulou *et al.*, 2008 ▸; Leban *et al.*, 2010 ▸; Revesz *et al.*, 2011 ▸; Xiao *et al.*, 2012 ▸; Muller *et al.*, 2012 ▸; Cheng *et al.*, 2013 ▸).

Photon beam position and angle instabilities at experimental stations are attributed to fluctuations of stored electron beam orbit and vibrational and thermal distortion of beamline optical components. The usual target for stability in the vertical plane is 10% of the beam size in position and angle (Hettel, 1989 ▸). Most of the available photon beam monitors are sensitive to the beam position only, and hence the name photon beam-position monitor (PBPM). However, the measured beam position is determined by both the source position and angle. A single PBPM does not provide independent information about the photon beam source position and angle (Tucoulou *et al.*, 2008 ▸; Kachatkou *et al.*, 2013 ▸). The photon beam angle also needs to be monitored to account for the negative effects of beam angle instability (Tucoulou *et al.*, 2008 ▸; Hahn *et al.*, 1998 ▸; Kyele *et al.*, 2007 ▸; Morse *et al.*, 2010 ▸). The use of two-photon PBPMs is common when the position and angle of a photon beam is to be measured (Tucoulou *et al.*, 2008 ▸; Muller *et al.*, 2012 ▸; Cheng *et al.*, 2013 ▸).

At the CLS, beam instabilities are monitored by two diagnostic beamlines: the Optical Synchrotron Radiation (OSR) beamline and the X-ray Synchrotron Radiation (XSR) beamline (Bergstrom & Vogt, 2006 ▸). Most beamlines at the CLS have provision for some type of PBPMs, but few are actually implemented or used. These monitors are of the type that measure only the photon beam position at some location in the beamline.

As with almost all synchrotron experiments, imaging is affected by photon beam motion. One of the imaging methods used at the BMIT beamline is *K*-edge subtraction using an iodine contrast element with a beam prepared by a bent Laue monochromator. Measurements made with this system during a period of electron beam instability gave the idea that we can measure the photon beam position and angle or from it infer the electron beam’s position in phase space.

We present a method for measuring the position and angle of a photon beam simultaneously, *i.e.* using a phase-space beam position monitor (ps-BPM). The method relies on the energy-dispersive properties of flat crystals and makes use of the absorption edge of a filter in the photon beam path to determine a specific energy or angle of the photon beam. This, coupled with a measurement of the beam in the absence of the filter, allows beam position and angle to be determined.

### 1.1. Synchrotron radiation   

**Figure 1:**
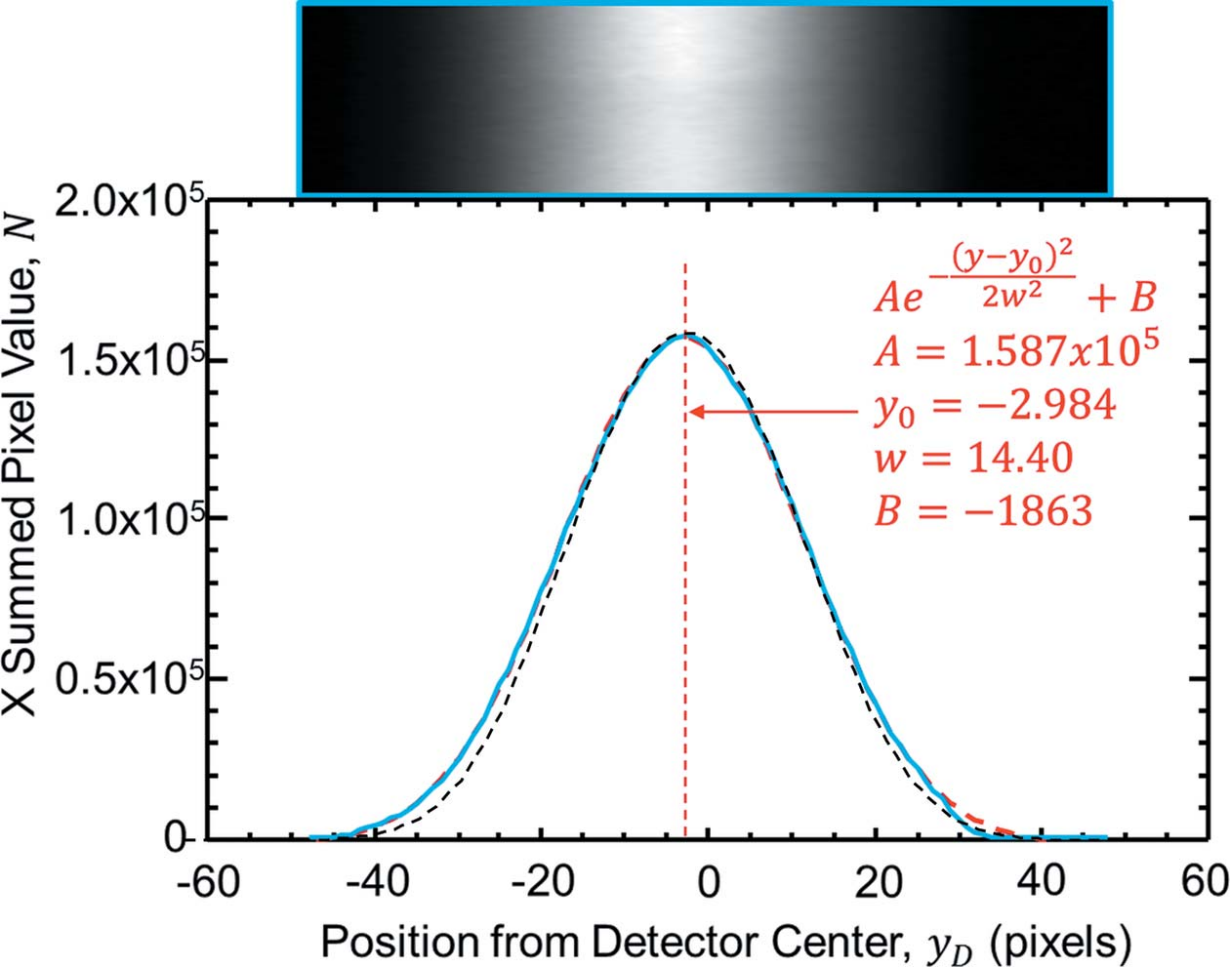
Nearly Gaussian vertical beam profile as measured on the CLS BMIT bend-magnet beamline. The picture at the top is an image of the beam. The plot at the bottom shows the measured beam profile from that image (blue) and a Gaussian fit (red dash). The red dotted line identifies the center. The red text gives the least-squares Gaussian fitting parameters. The vertical scale is in pixel units (100 µm pixel size). The calculated beam profile is shown as the black dashed line.

The single electron vertical photon emission distribution is properly described by a modified Bessel function of the second kind (Thompson *et al.*, 2009 ▸); however, this distribution is well modelled as a Gaussian function. This vertical angular distribution mostly falls within a 1/γ range in the X-ray regime where γ is the electron beam energy divided by the electron rest mass (γ = 5675 for the CLS). The electron beam size and vertical angle distribution can also be described as a Gaussian function. Therefore, the bend-magnet synchrotron beam has a vertical distribution that is nearly Gaussian. A measured comparison with theory for the vertical distribution of the beam on the CLS BMIT bend-magnet beamline is shown in Fig. 1 ▸ at 33.17 keV. As measured some distance from the source, the vertical angle or position motion of the electron source will move this distribution vertically. Because the measurement distance from the source can be tens of metres, the angle affects are amplified. For example, the profile measured in Fig. 1 ▸ is at 26 m from the electron beam source. In this figure the blue line is the measured profile from the image at the top of the figure. The red dashed line is a Gaussian fit to that profile. The fact that the red dashed line is difficult to see indicates how closely the Gaussian fit is to the actual beam profile. The black dashed line is a calculated profile from the modified Bessel function (Thompson *et al.*, 2009 ▸) for the actual conditions of the measurement [CLS bend magnet (2.9 GeV and 1.354 T) at 26 m from the source with 100 µm pixels and 33.17 keV photon energy].

### 1.2. Double-crystal monochromator at an absorption edge   

Diffraction of X-rays in crystals can be thought of as arising from constructive interference from reflections at lattice planes as described by Bragg’s law,
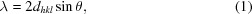
where λ is the wavelength of the diffracted beam, θ is the angle between the incident beam and lattice planes, and *d*_*hkl*_ (called the *d*-spacing) is the spacing between the *(h,k,l)* lattice planes.

For an X-ray synchrotron beamline, usually a pair of parallel crystals is used, one to monochromatize the incident beam and one to diffract that beam back parallel to the incident beam. With a coordinated motion of the two crystals, a range of energies can be chosen while keeping the monochromatic beam from the second crystal in the same location which is very useful for much of the research being done. Additionally, the near unit reflectivity of the perfect crystals often used means there is little intensity loss from the pair. This arrangement is commonly called a double-crystal monochromator (DCM) (Golovchenko *et al.*, 1981 ▸).

**Figure 2:**
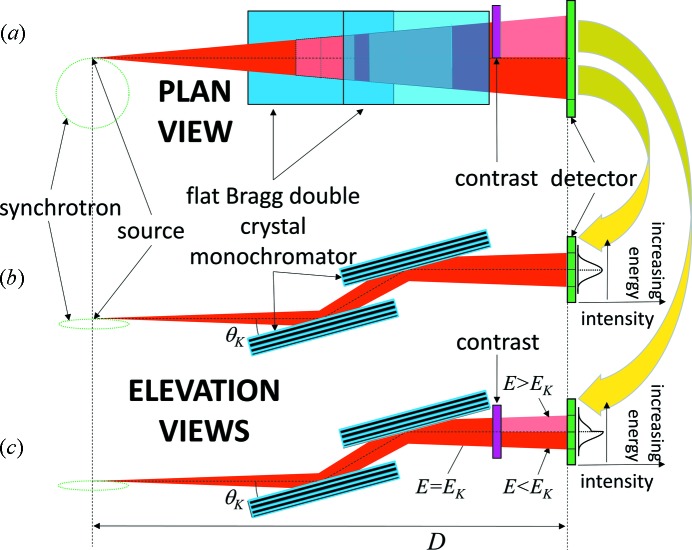
Schematic of the system used at the BMIT bend-magnet beamline. (*a*) Plan view of the double-crystal monochromator (DCM), contrast material and detector. (*b*) Elevation view of the non-contrast or beam side; (*c*) elevation view with contrast material whose *K*-edge is at the vertical middle beam prepared by the DCM. Example plots at the right show the profile for the unfiltered beam (*b*) and contrast filtered beam (*c*).

When a DCM is tuned to an energy, for example, the *K*-edge energy of an element, some of the transmitted beam will be above and some below the mean energy due to the dispersion properties of the crystals. This range of energies arises from the range of incident photon beam angles onto the lattice planes of the crystal and/or the energy bandwidth of the crystal and the reflection used. The range of energies due to angular divergence onto the planes is easily calculated using Bragg’s law based on the monochromator reflection used and the angular size of the beam passing through the system. A schematic of a DCM arrangement is shown in Fig. 2 ▸.

**Figure 3:**
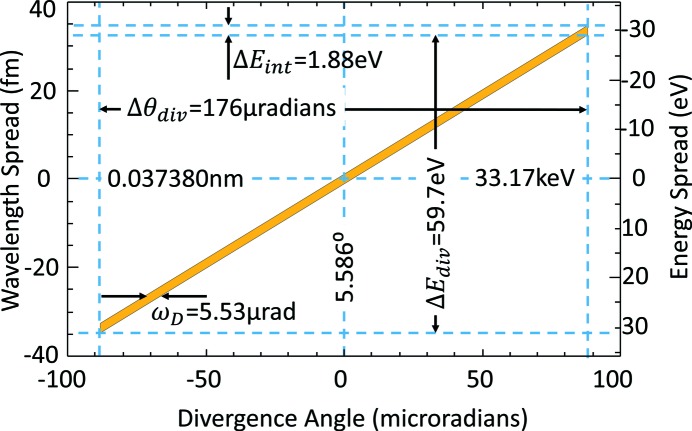
DuMond Diagram for Si (2,2,0) at 33.17 keV for 1/γ vertical divergence. The energy and angular ranges are shown for both vertical divergence and intrinsic widths. The relatively large vertical divergence results in an energy range that easily covers the *K*-edge of iodine.

As an example, assume a vertical angle range for the CLS of 1/γ = 176 µrad. At 33.17 keV, the absorption edge of iodine, the Bragg angle is 5.586° for the silicon (2,2,0) reflection. We can estimate the range of wavelengths using the derivative of Bragg’s law with respect to angle,
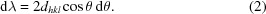
For the conditions stated, the wavelength spread is 6.73 × 10^−5^ nm centered at 0.03738 nm. The matching energy spread is 59.7 eV. Fig. 3 ▸ is a graphical representation, a DuMond diagram (DuMond, 1937 ▸), of Bragg’s law in the vicinity of the iodine *K*-edge.

There is also an energy or wavelength spread due to the finite reflectivity width of the dispersion curve. For diffraction, the energy or wavelength bandwidth is a fixed quantity away from absorption edges of the crystal. For silicon (2,2,0) the bandwidth is 56.6 × 10^−6^. Thus the intrinsic wavelength spread is 2.12 × 10^−6^ nm and the intrinsic energy spread is 1.88 eV. This wavelength and energy spread is also shown in Fig. 3 ▸. The energy spread due to divergence is almost 32 times that of the intrinsic energy spread.

**Figure 4:**
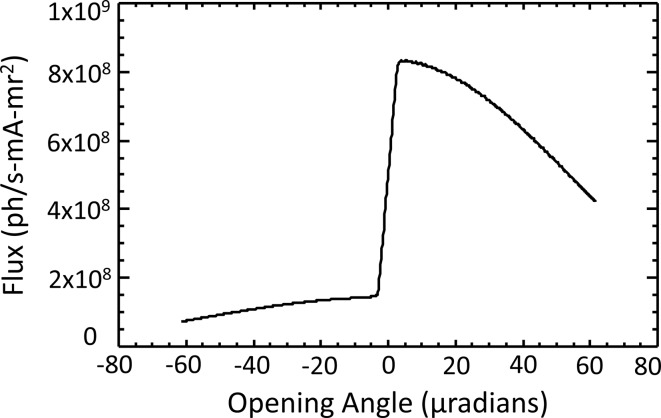
Calculated flux through a 60 mg cm^−2^ iodine filter from a Si (2,2,0) DCM at 33.17 keV on a CLS bend-magnet beamline.

Schematically, the effect of an iodine filter on the transmitted beam of a DCM set at 33.17 keV is shown in Fig. 2(*c*) ▸. Note that the spectral content of the beam vertically increases in energy from the bottom of the beam to the top. When the middle energy of the beam is placed at the iodine *K*-edge then the top of the beam will be absorbed more than the bottom creating an asymmetric beam profile shown on the right side of the figure. A calculation of the beam shape including the DuMond dispersion effects is shown in Fig. 4 ▸.

## 2. What happens when the beam moves?   

### 2.1. Unfiltered side of the beam   

**Figure 5:**
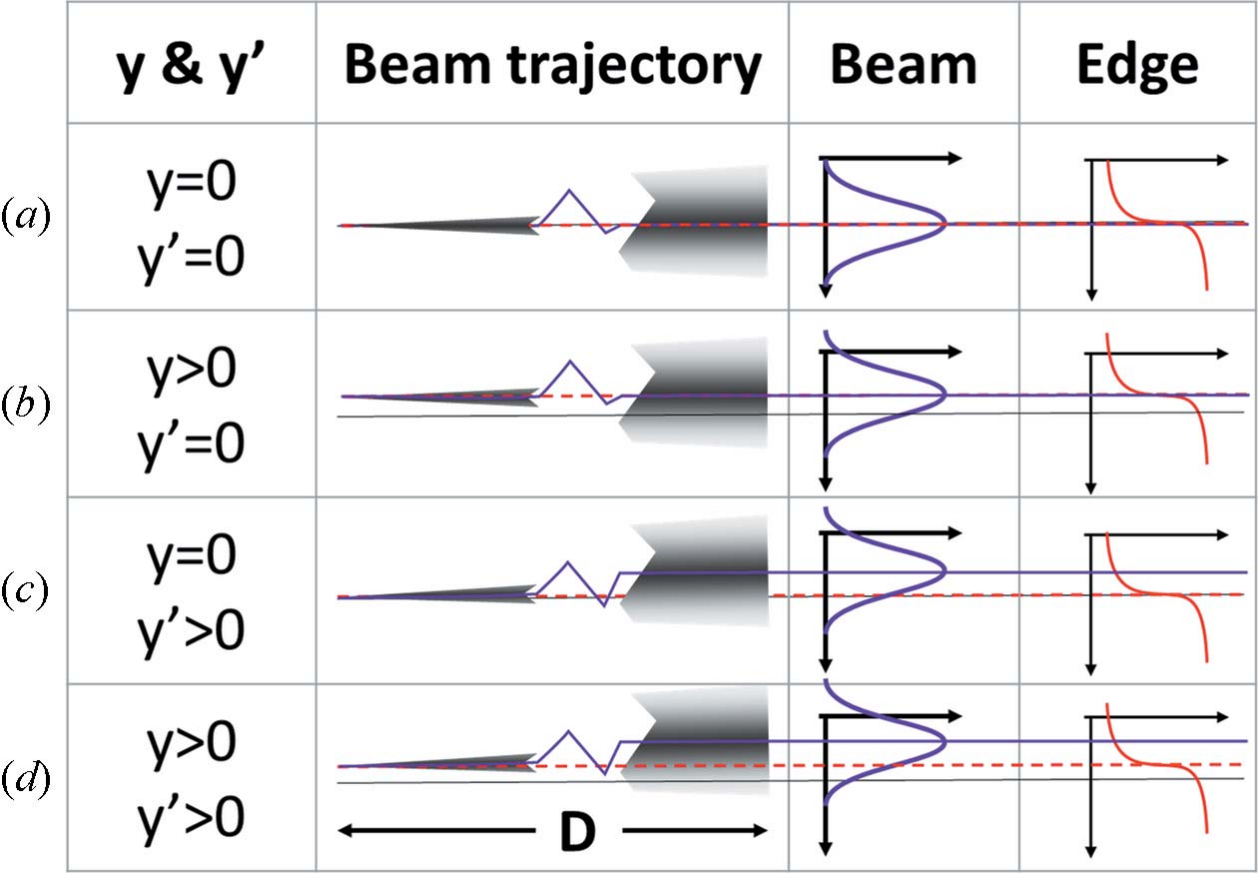
Schematic showing the effects of the electron beam position and angle displacements. The first column gives the position and angle, the second column is a schematic of the beam where the monochromator has been removed for clarity, the third and fourth columns show the beam and edge profile, respectively. The black line in each row represents the electron beam position and angle zeros, the purple line shows the centroid of the beam, the red dashed line shows the location of the same angle to the monochromator crystal or the edge location. (*a*, *c*) At *y* = 0. (*a*, *b*) At *y*′ = 0. (*b*, *d*) At *y* > 0. (*c*, *d*) At *y*′ > 0.

When a photon beam moves at the source location the monochromatic beam after the DCM is sensitive to the motion. If the source point moves up then the beam measured at the detector location will also move up by the same amount. If the beam moves in vertical angle then the beam at the detector position will move by the product of the angle times the distance from the source to the detector. This effect is shown schematically in Figs. 5(*a*)–5(*d*) ▸ in the ‘Beam’ column. The combination of vertical beam motion and angle is
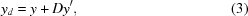
where 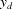 is the measured vertical beam position at the imaging detector, *y* is the vertical position of the electron beam source, 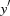 is the vertical angle of the electron beam source and *D* is the distance from the source to the detector.

### 2.2. *K*-edge filtered side of the beam   

The *K*-edge of an element is a fixed energy and can be used to locate that energy in the photon beam; energies above the edge will be heavily absorbed and energies below the edge will not. If the source moves vertically the location of the *K*-edge transition will move the same amount at the detector. This is because the vertical energy distribution of the photon beam is not altered by this motion. If instead the beam at the source moves in vertical angle the location of the edge will not move. In this case the vertical photon beam energy distribution is changed by the DCM but the vertical location of the *K*-edge at the detector will not move because the angle is set by the monochromator. Therefore the location of the *K*-edge at the detector is a direct measure of the location of the source vertically. This effect is shown schematically in Figs. 5(*a*)–5(*d*) ▸ in the ‘Edge’ column. The location of the *K*-edge measured at the detector is then simply
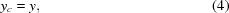
where *y*_*c*_ is the measured vertical *K*-edge location and *y* is the vertical position of the electron beam source. Changes in the vertical source angle do not change the location of the *K*-edge at the detector.

## 3. Determining the electron source vertical position and angle   

We are now in a situation to be able to independently determine the vertical electron beam position and angle by measuring the beam location through a DCM without a filter (beam side) and the edge location with a *K*-edge filter in place (edge side); using equations (3) and (4),
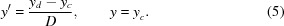
It should be noted that changing the monochromator energy has the effect of changing the source position, *y*_*c*_. Also, changing the detector’s vertical position will alter the *y*_*d*_ value. Both have a direct impact on the calculated electron beam position and angle, *y* and *y*′. Thus the system measures relative values of position and angle and must be calibrated to obtain absolute values.

The ability to determine the location of the beam centroid, *y*_*d*_, and the *K*-edge, *y*_*c*_, are integral to the success of this method. A fitting procedure will be used to determine the location of each. To be properly fit there needs to be sufficient intensity, detector resolution and vertical size to encompass the profile along with any vertical motion that may occur. The vertical beam size at the detector is a relatively weak function of the energy selected by the monochromator. However, the reflection chosen in the monochromator may have a strong effect on the intensity since the reflection sets the monochromator bandwidth.

In addition, the ability to determine *y*_*c*_ will also depend on the width of the *K*-edge and the thickness and density of the contrast filter. A rough estimate of the optimal projected iodine filter density was found to be ∼70 mg cm^−2^ by numerical simulation. This estimate was based on a contrast to noise model using Poisson statistics.

The width of the transmitted *K*-edge with the DCM will depend on the intrinsic *K*-edge width for the contrast element (∼15 eV for iodine; Feiters *et al.*, 2005 ▸). The *K*-edge will also be blurred by the intrinsic energy width of the monochromator [1.88 eV as discussed above for the Si (2,2,0) at 33.17 keV], and will also be dispersed vertically, *z*, across the detector, approximately as
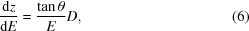
where θ is the Bragg angle, *D* is the source-to-detector distance and *E* is the *K*-edge energy. For silicon (2,2,0) at the iodine *K*-edge this spatial dispersion is 74 µm eV^−1^. Therefore, the 15 eV of energy spread will correspond to a spatial width of 1.1 mm.

When compared with silicon (4,4,0) under similar conditions the spatial dispersion at 33.17 keV will change to 150 µm eV^−1^ and the 15 eV energy spread will correspond to a width of ∼2.2 mm which indicates increased spatial dispersion sensitivity. But this sensitivity will come with a loss of intensity of over a factor of six due to the decreased (4,4,0) bandwidth (9.1 × 10^−6^) compared with the (2,2,0) bandwidth (56.6 × 10^−6^) and therefore, for the bulk of the measurements, we chose silicon (2,2,0).

## 4. Implementation at BMIT   

The experiments for this project were performed at the CLS BMIT bend-magnet beamline 05B1-1. A silicon (2,2,0) and (4,4,0) double-crystal monochromator was tuned to the iodine *K*-edge at 33.17 keV. The vertical dispersion of the monochromator allows an energy range that covers the *K*-edge of iodine. Figs. 2(*a*)–2(*c*) ▸ show schematically how the system was implemented in the beamline with a plan view at the top. In this system the beam was split horizontally in two parts: one side with a 60 mg cm^−2^ iodine filter and the other side with no filter. A Hamamatsu flat-panel detector with 0.1 mm pixel size was used to collect data. Measurements were made in the POE-2 hutch, which was ∼25 m away from the source.

Two types of measurements were performed. One type was made during the normal operational mode to assess the beam stability. A second type was made during special shifts where the synchrotron beam was intentionally moved at the source location with specific vertical, horizontal and angular offsets. This second type was used to assess, in part, the sensitivity of the system and to independently measure the motions made to the electron beam source in the ring.

Data were in the form of images of the split beam with the iodine filter on one side and no filter on the other. Sets of 400 data images were saved into individual directories and a measurement set might range from a few to several hundred directories. It took around 12 s to collect 400 images for each directory and roughly 1 min dead-time to save the data to the disk. For each set of measurements, ten dark images (the detector response without beam) and ten flat images (no contrast agent in the beam) were also collected for data normalization. These ten images were averaged to form single ‘dark’ and ‘flat’ images.

## 5. Data analysis method   

**Figure 6:**
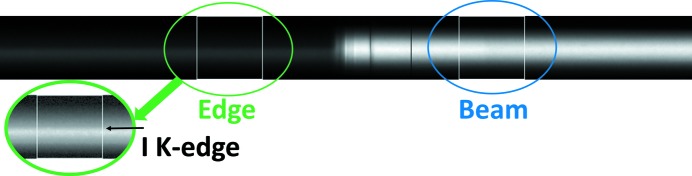
Example data image. Regions are chosen from both image types for beam and edge analysis. The edge region is enhanced in the lower left corner to better show the *K*-edge whose location is indicated by the arrow.

To analyze the data, several procedures were written in IDL (Interactive Data Language; ITT Visual Information Solutions, Boulder, CO, USA). Regions for the unfiltered beam side and the *K*-edge side were selected from the data and each side was corrected for dark response. An example of the regions chosen from the images is shown in Fig. 6 ▸.

The beam side data were used to determine *y*_*d*_ which was found by fitting the horizontally averaged vertical beam profile using a Gaussian function. An example of this fitting was shown in Fig. 1 ▸. The vertical direction at the detector, *z*, was measured in terms of detector pixels that can be easily converted into micrometers using the pixel size. The vertical center of the detector was the origin used in this part of the analysis.

**Figure 7:**
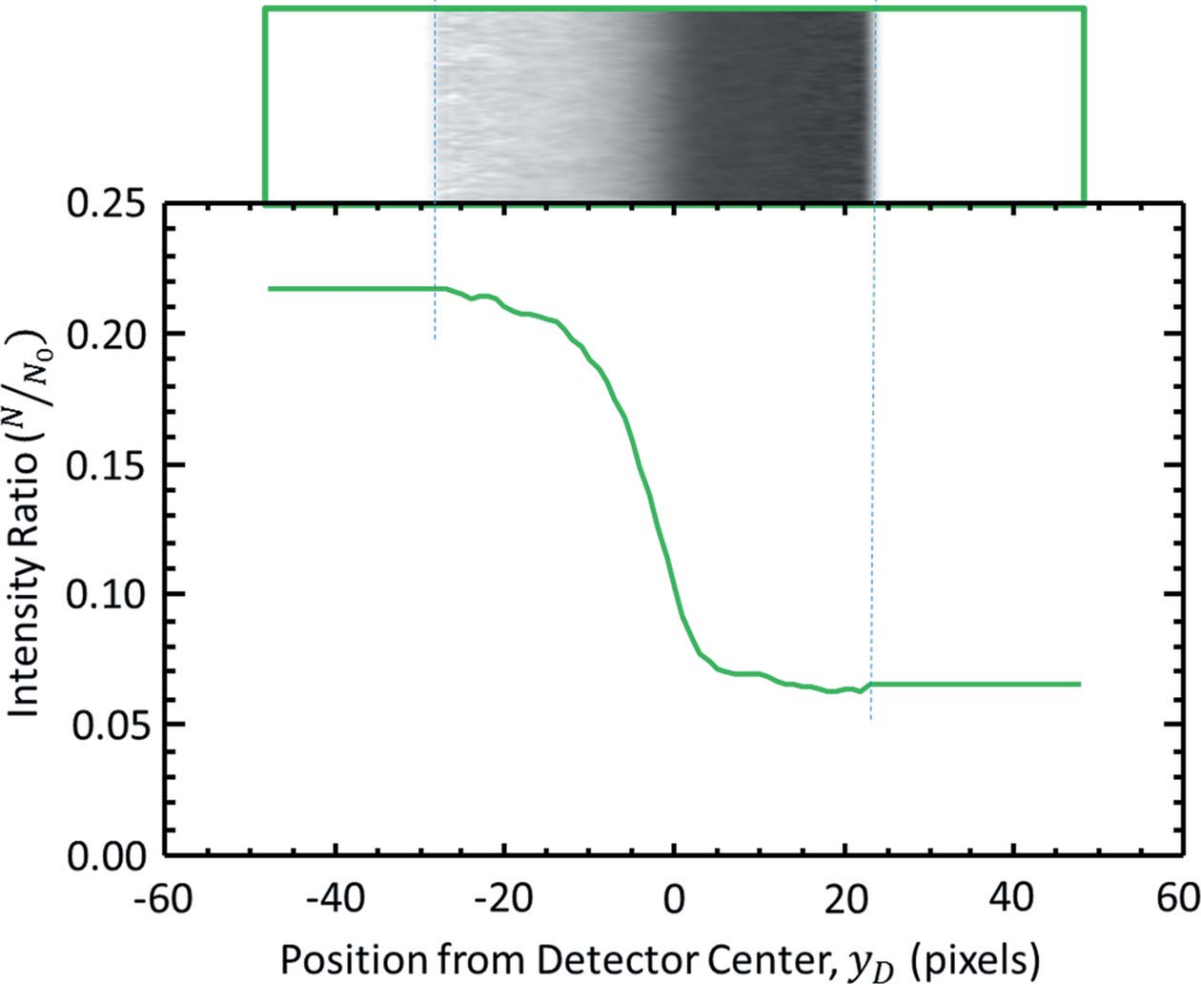
Summed normalized *K*-edge image across the sampling width; 100 pixels in this case. The horizontal axis is in pixels and the origin is referenced to the vertical middle of the detector.

The horizontally averaged *K*-edge side profile was normalized by the matching region from the flat to form an *I*/*I*_0_ profile. However, the flat was corrected for vertical beam motion by using the beam side of the data to vertically move the flat prior to normalization. The low-intensity regions of this corrected flat were ignored using a threshold value and not used in the analysis. An example of this is shown in Fig. 7 ▸.

**Figure 8:**
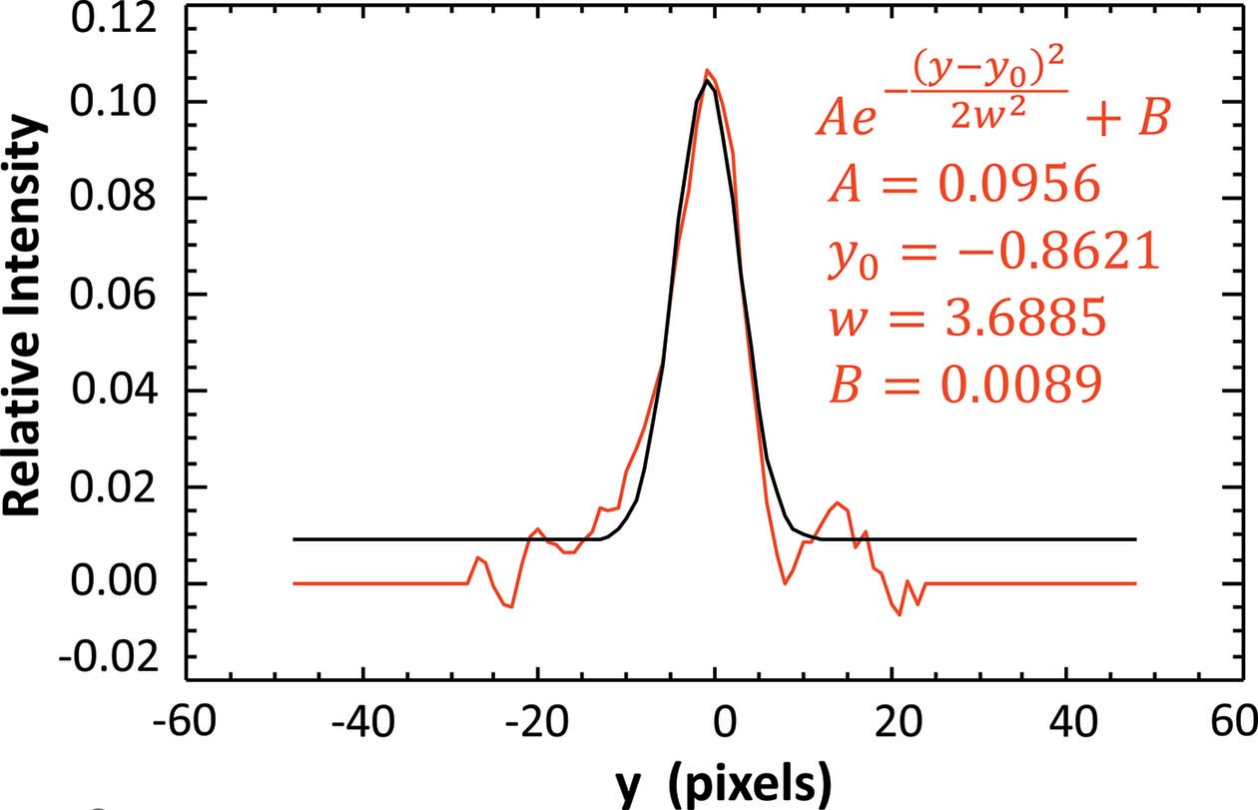
Derivative of the negative logarithm of the profile shown in Fig. 7 ▸. The Gaussian fit parameters are shown in the upper right-hand corner. For this analysis only *y*_0_ or the peak center is used.

A number of fitting algorithms were used to find the location of the *K*-edge in the filtered beam profile. In general, three types of functions were investigated to fit the edge: Gaussian, Lorentzian and Voigt. These functions were chosen because they are mathematically simple and make physical sense. The functions were used directly to fit the derivative of the *K*-edge profile, and integrated versions of each were used to fit the measured edge profile directly. Finally the *K*-edge could be fit to the *I*/*I*_0_ values or the negative logarithm of the *I*/*I*_0_ values. Of the 12 fit types the Gaussian fit to the derivative of the negative logarithm of the profile was chosen due to its simplicity and the robustness of the fit. For this analysis the main parameter of interest in the fit was the transition center location. Other parameters determined in the fit were the amplitude, width and the background values. Fig. 8 ▸ shows a fit to one of the data points.

## 6. Results and discussion   

A number of measurements have been taken with the system. In total, eight shifts (1 shift = 8 h) were used for the beam motion measurements. These shifts were during normal operations and by special request when the ring parameters could be altered. During the special machine study shifts, the electron beam was moved and measurements were made to assess the response of the system.

### 6.1. System response to electron beam motions   

**Figure 9:**
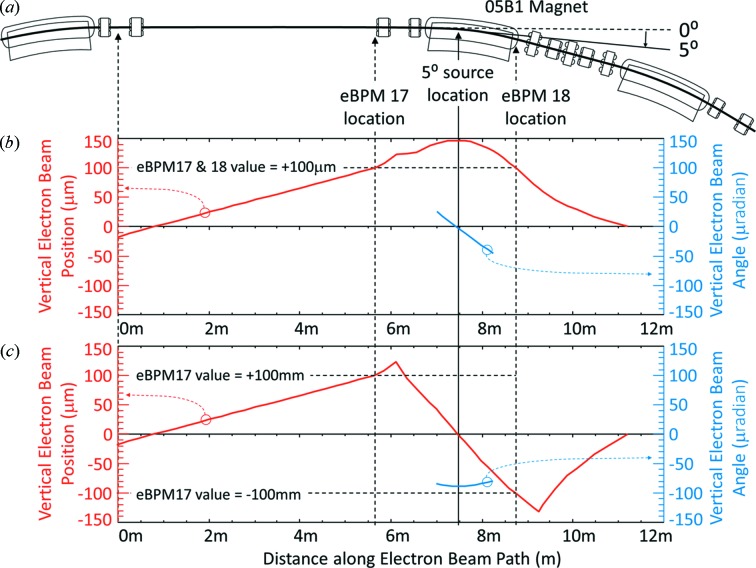
Storage ring schematic and calculated electron beam trajectories. (*a*) Section of the storage ring around the 05B1 magnet from which the measurements were made. The locations for eBPM17, eBPM18 and 5° source are indicated. The calculated trajectory for +100 µm vertical position for eBPM17 and eBPM18 are shown in (*b*) with the electron vertical position in red and angle in blue. (*c*) Trajectory for a +100 µm value at eBPM17 and −100 µm at eBPM18 which mostly creates an angle at the source location.

The electron beam position and angle were varied in the machine study shifts. To move the electron beam in the BMIT sector, electron BPM (eBPM) target values were changed. This change in target value moves the electron beam orbit using dipole steering magnets. The eBPMs numbered 17 and 18 are located on the upstream and downstream sides of the bend magnet which is our photon beam source (see Fig. 9 ▸). The photon beam source is 5° into the 15° bend. Since we observe the photon beam source point at an intermediate location between the two eBPMs, there can be small differences in the electron beam vertical position and angle due to the storage ring magnetic optics.

#### 6.1.1. Electron beam vertical position measurements   

**Figure 10:**
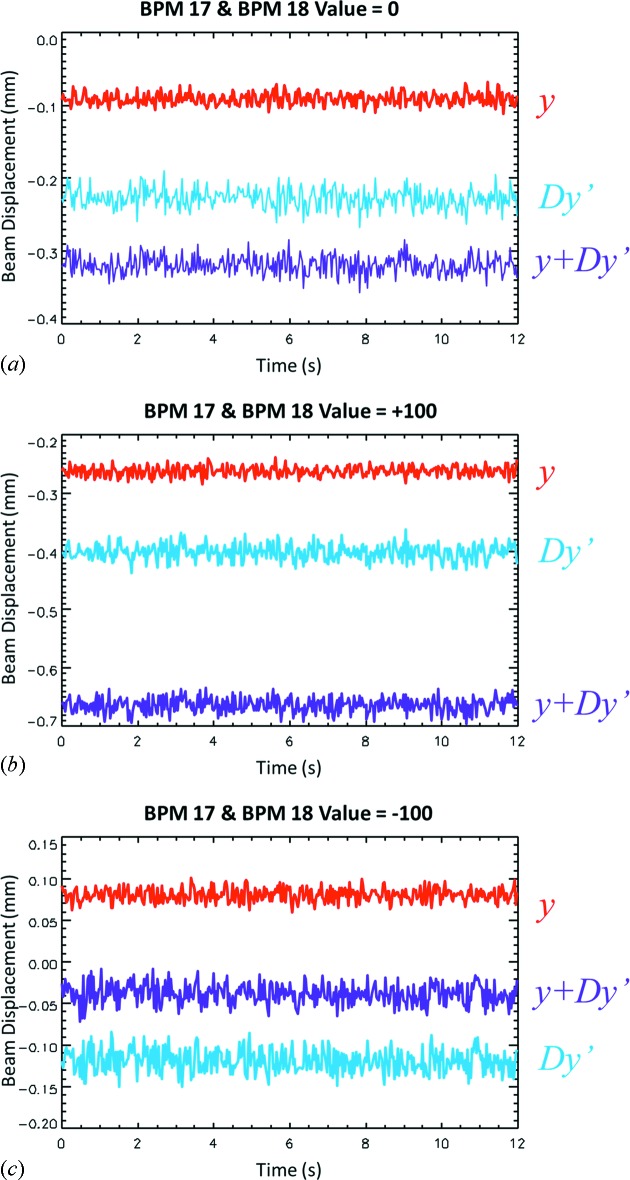
Measurements of the beam vertical position, *y*, the effect of vertical angle, *Dy*′, and combined motion as a function of time for eBPM17/18 values of 0 (*a*), +100 (*b*) and −100 (*c*). The vertical motions have been translated into millimeters using the 100 µm pixel size. The vertical zero is the vertical detector center.

For vertical motion, the eBPM17 and eBPM18 target values were changed by the same amount. The beam was moved vertically between +100 and −100 µm in 0, ±1, ±2, ±5, ±10, ±20 and ±100 µm increments. Examples are shown where the beam is at the zero location (Fig. 10*a*), +100 µm (Fig. 10*b*) and −100 µm (Fig. 10*c* ▸). In those plots, 12 s of beam motion is shown. It should be noted that the measured beam position and angle are relative to the middle of the detector. Three data lines are shown: red, which is the measured electron beam position, *y*; blue, the electron beam angle multiplied by the distance from the source to the detector, *Dy*′ (this allows it to be plotted on the same scale as the position); and purple, the overall beam position, *y* + *Dy*′.

**Figure 11:**
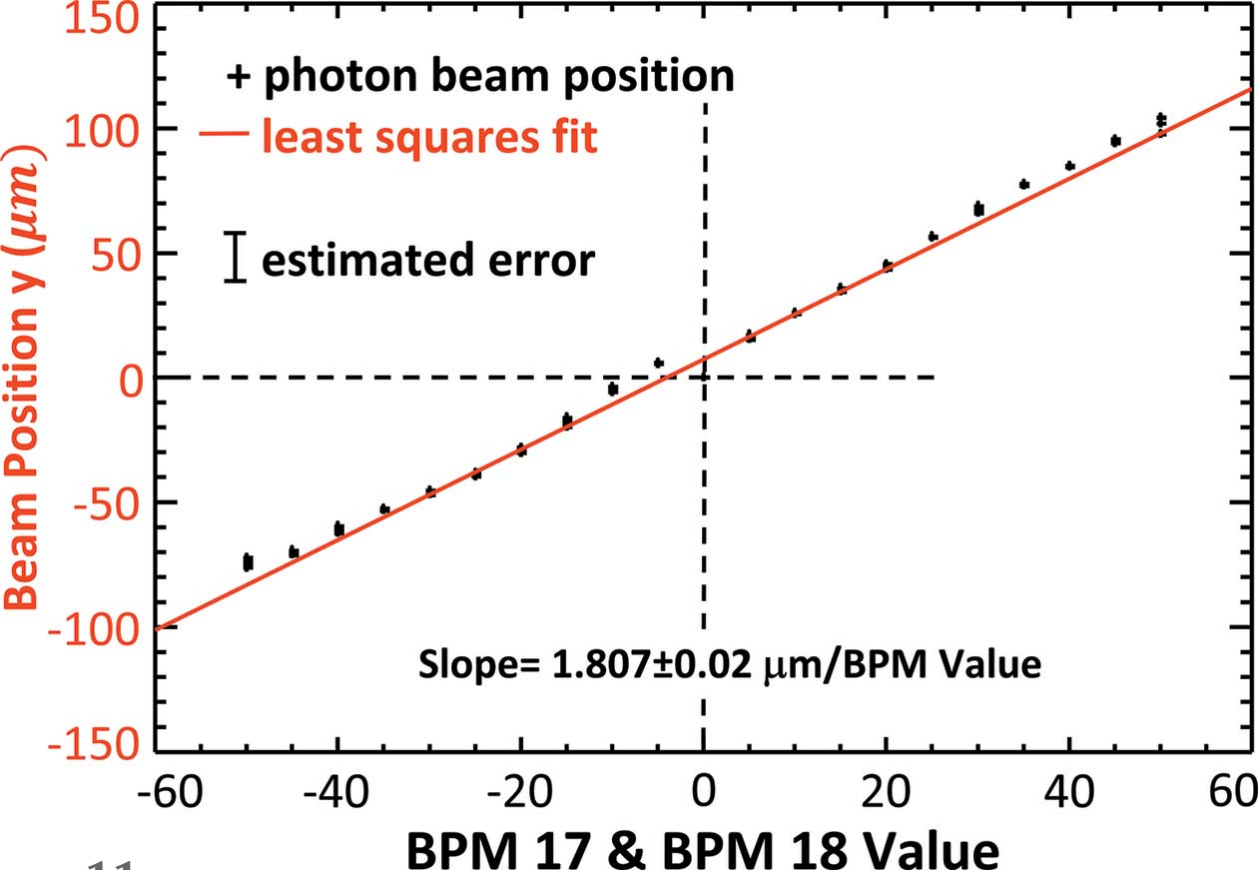
Electron vertical beam position in micrometers measured as the eBPM17 and 18 are changed from −50 to +50 µm.

**Figure 12:**
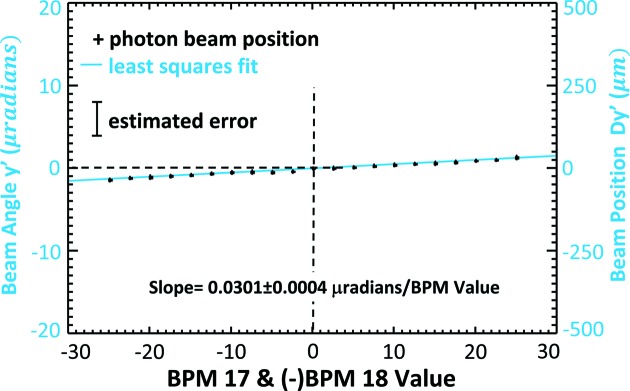
Electron beam vertical angle in microradians measured as the eBPM17 and 18 are changed from −50 to +50 µm. The measured angle in microradians is shown on the left axis and the vertical displacement that angle creates at the detector position, *Dy*′, is shown on the right.

Trend plots of the electron beam position and angle measurement as a function of eBPM offset values are shown in Figs. 11 ▸ and 12 ▸, respectively. Some selected values are also given in Table 1 ▸. The dashed horizontal line in the figures identifies the measured ‘zero’ location where the electron beam is at the zero location in eBPM units. Clearly, there is good correlation between the eBPM values and the beam position and angle. The red line in Fig. 11 ▸ and the blue line in Fig. 12 ▸ are least-squares fits to the measured data.

For these data, 150 twelve-second measurements (called a ‘slice’) were made as the beam was moved. For each beam location, between four and five slices were taken. In reviewing Table 1 ▸, the measured standard deviations are in the 10 µm range for *y*, *Dy*′ and *y* + *Dy*′.

From Fig. 11 ▸, the vertical beam position rate of change was determined to be 1.807 ± 0.02 µm per µm eBPM value from the least-squares fit. From Fig. 12 ▸, the vertical beam angle rate of change was similarly determine to be 0.0301 ± 0.0004 µrad per µm eBPM value. Based on electron beam optics calculations for an ideal machine, the predicted values should be 1.47 µm per µm eBPM value and −0.034 µrad per µm eBPM value, respectively. The good agreement between the experimentally determined and calculated values is shown in the top row of Table 2 ▸.

#### 6.1.2. Electron beam vertical angle measurements   

Similar measurements were made when the eBPM values were changed asymmetrically to create electron beam angle at the beamline. Tests were made with eBPM offsets between +20 and −20 µm in 0, ±5, ±10, ±15 and ±20 increments.

**Figure 13:**
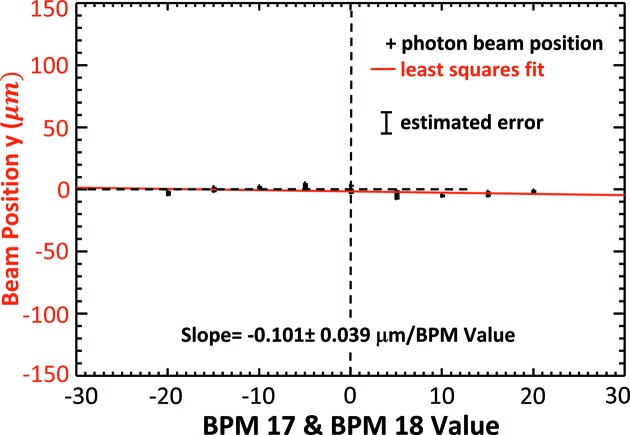
Electron vertical beam position in micrometers measured as the eBPM17 and −18 are changed from −20 to +20 µm.

**Figure 14:**
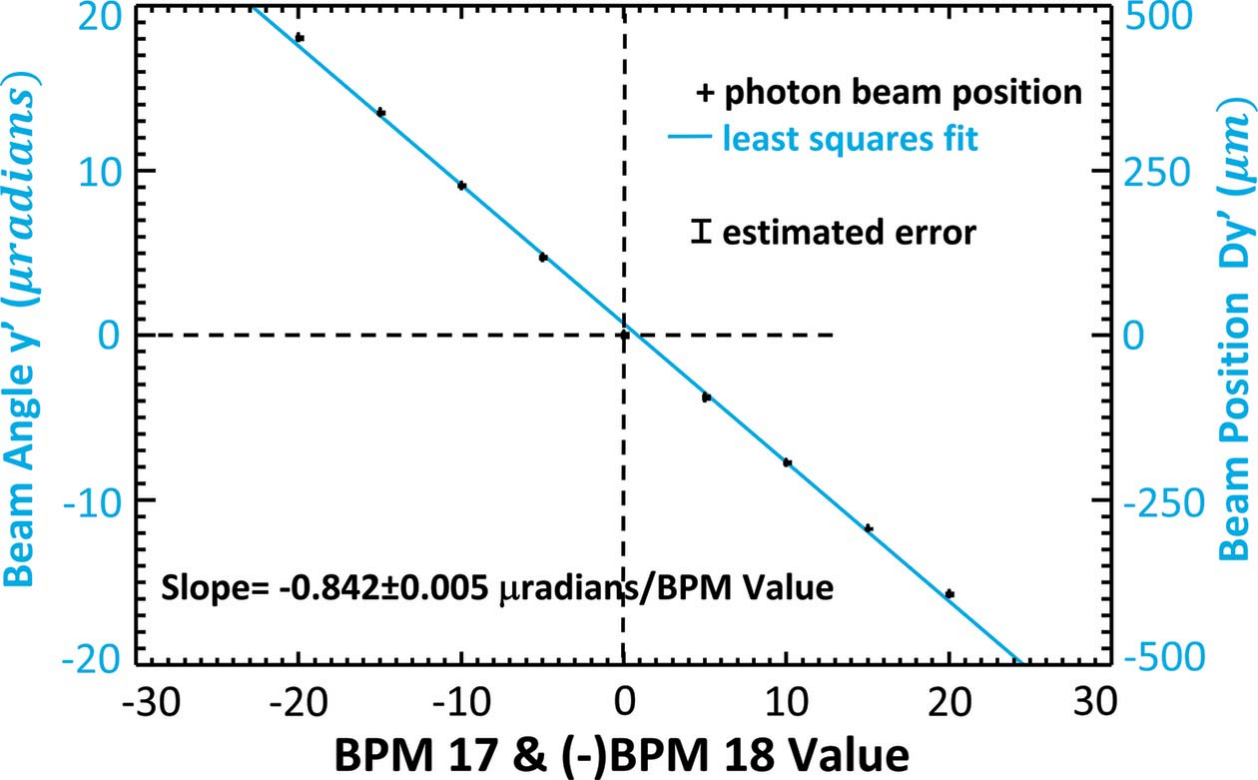
Electron beam vertical angle in microradians measured as the eBPM17 and −18 are changed from −20 to +20 µm. The measured angle in microradians is shown on the left axis and the vertical displacement that angle creates at the detector position, *Dy*′, is shown on the right.

Plots of the electron beam position and angle as a function of eBPM values are shown in Figs. 13 ▸ and 14 ▸, respectively. Some selected values are also given in Table 3 ▸ and, as before, the measured standard deviations are in the 10 µm range for *y*, *Dy*′ and *y* + *Dy*′. The dashed horizontal line in the figures identifies the measured zero location where the electron beam is at the zero location in eBPM units. The red line in Fig. 13 ▸ and the blue line in Fig. 14 ▸ are least-squares fits to the measured data.

The measured and calculated beam responses are summarized in the lower half of Table 2 ▸. Again, there is a good agreement between the measured and ideal machine values.

### 6.2. Normal operations measurements   

Measurements with the ps-BPM system were made during a number of normal operation shifts between December 2013 and August 2014. During the December 2013 to early 2014 period the CLS storage ring was experiencing beam instabilities from a storage ring dipole magnet power supply.

**Figure 15:**
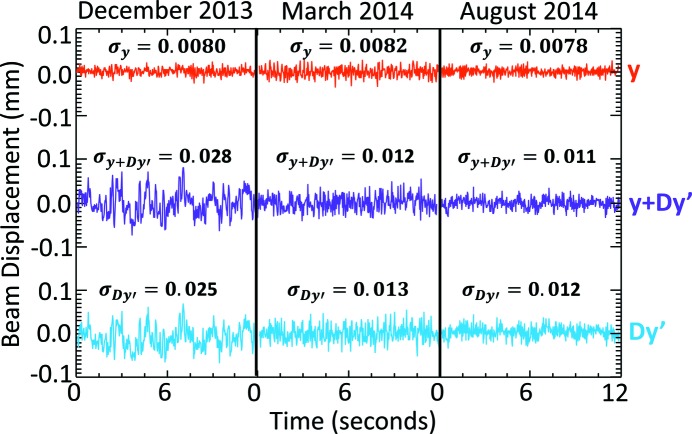
Beam phase-space measurements over a 12 s interval during normal operations for three dates: December 2013, March 2014 and August 2014. The top line is the zero referenced electron beam position, *y*, the bottom line is the vertical displacement due to angle, *Dy*′, and the middle line is the sum of the two, *y* + *Dy*′. Note the improvement in beam stability over the nine-month period. The standard deviation values are shown above each period.

Fig. 15 ▸ shows 12 s measurements made during three operational periods in December 2013, March 2014 and August 2014. The top line is the measured electron beam position, *y*, the bottom line is the vertical displacement due to angle, *Dy*′, and the middle line is the sum of the two, *y* + *D* *y*′. Each of the three measurement periods are shown side by side in the figure. The offsets for the three position types have been removed so that their average is zero to make the comparison easier.

It is clear that the ring stability improved dramatically during that time period and that the instability measured arose primarily from the angle displacements. Fig. 15 ▸ also shows measured standard deviations of the positions, 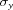, 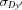 and 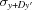. The standard deviation of the electron beam position, 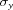, varied little over the three periods. However, the standard deviation of the vertical displacement due to angle, 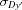, dropped by a factor of two in the same time. The standard deviation of the overall beam displacement due to electron beam positon and angle, 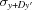, is determined mostly by the electron beam angle.

The DCM could be responsible for some of the measured beam motion due to vibrations and thermal motion. Internal vibrations within the monochromator are somewhat minimized as we used a gravity flow water system for cooling both crystals in the DCM. Also the use of a copper and aluminium filter dropped the incident power on the first crystal to below 2 W for the size of the beam and ring current used for the experiments. Finally, based on our measurements, as the beam is moved in the machine the error bar on the beam displacement was typically less than 10 µm and the angle less than 0.5 µrad, indicating that the combination of the monochromator and electron beam motions must be less than these values.

## 7. Practical implementation of a ps-BPM   

All of the proof-of-principle measurements required the use of the entire beamline and as such would be completely impractical as a monitor. Probably the most challenging aspect of implementing such a monitor would be the dedicated use of the DCM. The complexity of the DCM in which the two crystals must be maintained in sub-microradian alignment in the Bragg geometry could be mitigated by the use of a single-crystal Laue or transmission-type monochromator. The Laue-type monochromator is not so susceptible to crystal heating effects as there is no thermal bump on the crystal’s surface. Much of the power can be transmitted through where it can be absorbed elsewhere. The width of the beam being used need not be very wide as two line detectors can be used for the beam and *K*-edge side measurements. A prototype Laue-type system is being designed.

## 8. Conclusions   

A unique method for simultaneously measuring the vertical position and angle (phase space) of synchrotron photon beams using the combination of X-ray diffraction and absorption edges has been developed and tested at the BMIT beamline at the Canadian Light Source. This system allows the measurement of the photon beam centroid in phase space, and thus the electron beam source position and angle, at a single location along the beamline. Temporal stability and beam motion system response measurements have been performed with good results.

The sensitivity of this system is comparable with other photon beam position monitors with detectable position errors on the scale of 10 µm and angle errors of 0.5 µrad. These errors are based on the noise level in a 12 s time measurement with 400 time points.

We are optimistic that a compact dedicated system employing a single transmission or Laue monochromator coupled with a *K*-edge filter and two line detectors can be built and implemented to make a practical device that uses a small piece of the horizontal width of a bend-magnet or wiggler white beam.

Such a phase-space system provides a more complete view of beam motion in a synchrotron source and may be used to improve the synchrotron source position and angle stability. The ps-BPM also provides a better diagnostic should instabilities or beam drift occur, and could be used to correct experimental data for beam motion and to actively control the trajectory of the photon beam in the beamline.

## Figures and Tables

**Table 1:** Selected measured electron vertical beam position, *y*, and angle, *y*, as a function of vertical electron beam motion defined by equal eBPM17 and 18 values The slice number identifies the measurement. Columns showing the effect of the electron beam angle on beam position, *Dy*, and the overall vertical beam motion, *y* + *Dy*, at the detector, are given along with calculated standard deviations. Each slice corresponds to 12s of acquisition time.

Slice #	BPM 17/18 (m)	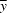 (m)	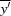 (rad)	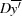 (m)	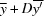 (m)
2	0	401 8	5.89 0.54	146 13	255 12
82	5	385 8	6.01 0.56	150 14	235 12
95	10	375 8	6.07 0.52	152 13	223 13
92	20	356 8	6.34 0.53	158 13	198 12
99	30	334 8	6.64 0.55	165 14	168 13
105	40	316 8	7.02 0.54	175 14	141 13
113	50	297 8	7.34 0.53	190 13	107 12
121	5	396 8	5.45 0.51	136 13	259 13
125	10	406 8	5.34 0.52	133 13	272 12
133	20	430 8	5.24 0.55	131 14	299 12
139	30	448 8	4.97 0.53	124 13	324 12
146	40	461 8	4.52 0.52	113 13	348 12
153	50	467 8	4.16 0.53	103 13	372 11

**Table 2:** Measured and calculated detector response to vertical electron beam position and angle Vertical electron beam positions where eBPM17 and 18 are equal are shown in the upper two rows (upper row: measured; lower row: calculated). Vertical beam angle where eBPM17 is equal to, but opposite sign to, eBPM18 is shown in the bottom two rows. At the measurement location there is a mixture of position and angle for both types of electron beam motion.

		Position 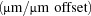	Angle 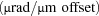
Position motion (eBPM17 = eBMP18)	Measured	1.807 0.02	0.0301 0.0004
	Calculated	1.47	0.034
Angle motion (eBPM17 = eBPM18)	Measured	0.101 0.039	0.842 0.005
	Calculated	0.06	0.88

**Table 3:** Selected measured electron vertical beam position, *y*, and angle, *y*, as a function of electron beam angle defined by equal and opposite sign eBPM17 and 18 values; the remainder of the table is for *Dy* and *y* + *Dy* as defined in Table 1 ▸ and in the text

Slice #	BPM 17/18 (m)	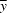 (m)	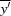 (rad)	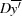 (m)	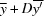 (m)
9	5	428 8	2.30 0.55	57 14	371 14
13	10	430 8	1.64 0.51	41 13	471 13
20	20	427 8	9.63 0.51	241 13	667 12
47	5	421 8	10.70 0.57	267 14	154 13
51	10	423 8	15.09 0.51	377 13	46 13
58	20	428 8	24.16 0.58	604 15	176 13
